# Coaching: a promising way to enhance implementation of best-practice methods

**DOI:** 10.1186/1940-0640-7-S1-A62

**Published:** 2012-10-09

**Authors:** Fredrik Spak, Per Blanck

**Affiliations:** 1 Department of Social Medicine, Gothenburg University, Gothenburg, Sweden

## 

Between 2006-2011, we interviewed implementation coaches responsible for educating primary health-care (PHC) providers in Western Götaland (1.5 million inhabitants) in new working routines concerning alcohol screening and brief intervention (SBI) as part of the Swedish Risk Drinking Project (RDP). The targets groups for SBI education were PHC staff responsible for roughly 100 family health-care (FHC) units and about 100 maternity health-care (MHC) and child health-care (CHC) units. Forty thematic interviews were conducted mainly by telephone with project leaders, coaches, midwives, nurses, and general practitioners (GPs). The interviews were transcribed, and their content was analyzed. On the positive side, the coaches created a good basis for establishing SBI in PHC settings. Staff in MHC and CHC settings accepted SBI as an important component of their work and increased their self-efficacy in, and routines concerning, SBI. The staff felt supported by the SBI coaches. For GPs, a positive change was noted for some in FHC practices in that more patients were asked about alcohol consumption, and interest in working on lifestyle changes increased. On the negative side, it was not clear if the positive changes were due to RDP coaching or some other factor. Also, the coaches were not given adequate time to work with FHC staff. Administrative changes over the course of the project, including partial privatization, resulted in a lower priority being given to SBI training and implementation. Also, GPs are still skeptical about systematic screening. All the groups interviewed expressed uncertainty on how to define risk drinking, and a common policy is lacking on this matter. New practices can be difficult to maintain if the relative utility of the enterprise seems small. We advocate a larger involvement of men in FHC preventive work, which has heretofore largely been undertaken by women.

